# Adaptive Epitaxy of C‐Si‐Ge‐Sn: Customizable Bulk and Quantum Structures

**DOI:** 10.1002/adma.202506919

**Published:** 2025-06-11

**Authors:** Omar Concepción, Ambrishkumar J. Devaiya, Marvin H. Zoellner, Markus A. Schubert, Florian Bärwolf, Lukas Seidel, Vincent Reboud, Andreas T. Tiedemann, Jin‐Hee Bae, Alexei Tchelnokov, Qing‐Tai Zhao, Christopher A. Broderick, Michael Oehme, Giovanni Capellini, Detlev Grützmacher, Dan Buca

**Affiliations:** ^1^ Peter Gruenberg Institute 9 (PGI‐9) and JARA‐Fundamentals of Future Information Technologies Forschungszentrum Juelich 52428 Juelich Germany; ^2^ IHP–Leibniz Institut für innovative Mikroelektronik 15236 Frankfurt (Oder) Germany; ^3^ Institute of Semiconductor Engineering University of Stuttgart 70569 Stuttgart Germany; ^4^ CEA LETI Grenoble 38000 France; ^5^ School of Physics University College Cork Cork T12 YN60 Ireland; ^6^ Tyndall National Institute Lee Maltings Dyke Parade University College Cork Cork T12 R5CP Ireland; ^7^ Dipartimento di Scienze Università degli Studi Roma Tre Roma 00146 Italy

**Keywords:** C(Si)GeSn alloys, epitaxial growth, multi quantum wells, RP‐CVD

## Abstract

The successful demonstration of (Si)Ge_1‐x_Sn_x_ alloys as direct‐gap materials for infrared lasers has driven intense research on group IV‐based devices for nanoelectronics, energy harvesting, and quantum computing applications. The material palette of direct‐gap group‐IV alloys can be further extended by introducing carbon to fine‐tune their structural and electronic properties, significantly expanding their functionality. This work presents heteroepitaxial growth of C(Si)GeSn alloys using an industry‐standard reduced‐pressure chemical vapor deposition reactor. The introduction of CBr_4_ as a precursor enables controlled incorporation of C atoms (<1 at.%) into the epilayer lattice, while simultaneously increasing the Sn content in the CGeSn alloy up to ≈18 at.%. Carbon plays a key role in modulating strain, stabilizing the crystal structure, and influencing material properties. By leveraging alloying and strain engineering, quaternary CSiGeSn bulk layers and CGeSn/GeSn heterostructures are epitaxially grown. The impact of C incorporation on optical emission is investigated in LEDs based on CGeSn/GeSn multiple quantum wells, demonstrating enhanced near‐infrared emission at 2.54 µm, which is sustained up to room temperature.

## Graphical abstract

This work pushes the boundaries of epitaxy by achieving all‐group‐IV semiconductor heterostructures and extensive characterization. The alloys open new research topics in material properties investigation for next‐generation electronic and optoelectronic device technologies.

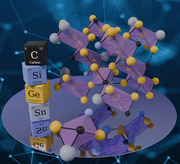

## 1 Introduction

The pursuit of advanced semiconductor materials has been central to technological progress since the mid‐20th century, fueled by the continuous demand for faster, more compact, and energy‐efficient Complementary Metal‐Oxide‐Semiconductor (CMOS) electronic devices. A key advantage of novel CMOS‐compatible materials such as CrGeTe_3_^[^ ^1^, ^2^ ^]^ and specifically solely group‐IV semiconductor technologies lie in their ability to extend on‐chip functionality. In this respect, the development of silicon photonics (SiP) has significantly expanded the Si‐microelectronic technological platform, whose functionalities have been further expanded by the integration of germanium‐based modules, enabling efficient on‐chip light waveguiding,^[^ ^3^ ^]^ modulation, and photodetection.^[^ ^4^, ^5^ ^]^ In the same vein, the scientific communityis dedicating significant effort to develop an efficient on‐chip silicon‐based light source, a critical component needed to complete the monolithic SiP toolbox.^[^ ^6^, ^7^, ^8^ ^]^

Another crucial functionality still missing from the Si‐CMOS platform is on‐chip heat management – essential for energy harvesting, device cooling, and temperature control – where II‐VI materials have traditionally excelled,^[^ ^9^ ^]^ at least at on‐chip temperatures. Unfortunately, this class of materials, which includes PbTe, poses health and environmental concerns and is not compatible with the Si‐CMOS platform. Recently, GeSn alloys have emerged as a promising alternative^[^ ^10^, ^11^ ^]^ as CMOS‐compatible thermoelectric materials.

The incorporation of Sn into Ge and SiGe matrix to form binary GeSn or ternary SiGeSn alloys has emerged as a promising avenue for further material innovation.^[^ ^12^, ^13^ ^]^ Indeed, the addition of Sn offers the possibility for bandgap and strain engineering, enabling, e.g. the realization of direct bandgap, CMOS‐compatible group‐IV semiconductors, which are critical for the development of the next generation of devices. The Sn‐based system has already been exploited to realize high‐performance nanowire FETs,^[^ ^14^, ^15^ ^]^ photodetectors,^[^ ^16^ ^]^ lasers,^[^ ^17^, ^18^ ^]^ and LEDs,^[^ ^19^, ^20^, ^21^ ^]^ as well as to demonstrate suitable properties for spintronics^[^ ^22^ ^]^ applications.

The technological breakthrough required to enable monolithic integration of photonic, electronic, thermoelectric, and spintronic functionalities – paving the way for complex, energy‐efficient integrated circuits – can only be achieved using semiconductors based on group‐IV elements: C, Si, Ge, and Sn. The incorporation of C to realize quaternary C(Si)GeSn alloys can offer even broader scope for bandgap engineering,^[^ ^23^, ^24^ ^]^ and heterostructure design to deliver enhanced properties,^[^ ^25^, ^26^ ^]^ potentially unlocking novel functionalities.

However, considering that the epitaxial growth of ternary (Si)GeSn alloys is already highly challenging due to the metastable nature of these compounds,^[^ ^27^, ^28^, ^29^ ^]^ further incorporating C introduces a significant level of complexity. Carbon has an even lower solid solubility in Ge (<0.1 at.%) compared to Sn (<1 at.%), and its covalent radius (0.077 nm) is much smaller than Ge (0.122 nm) and Sn (0.141 nm). As a result, C atoms in C(Si)GeSn alloys are prone to precipitate or migrate into interstitial positions during growth and/or post‐processing. To alleviate these challenges requires precise control over the growth parameters and material processing, to stabilize C incorporation and harness its potential benefits in these quaternary group‐IV alloys.

Despite recent reports of CGeSn growth on GaAs^[^ ^30^, ^31^ ^]^ via molecular beam epitaxy (MBE), or earlier reports of growth on Si substrates via radio‐frequency magnetron sputtering,^[^ ^32^ ^]^ no CMOS‐compatible or industry‐transferable chemical vapor deposition epitaxy studies are available to date. Even though the Sn content in those layers is relatively low (< 8 at.%) and the crystalline quality of the samples is relatively poor, reported absorption spectra confirm the potential to tune the bandgap via C incorporation, highlighting the material's potential for bandgap engineering.

To progress toward integration of quaternary alloys into the CMOS platform, in this work, we report the epitaxial growth of CGeSn and CSiGeSn semiconductors on 200 mm Si(001) substrates using an industry‐standard reduced‐pressure chemical vapor deposition (RP‐CVD) method. We examine the role of key growth parameters, including precursor partial pressures and growth temperature, on the alloy composition. A thorough investigation of the structural and optical properties highlights the role of C atoms in governing heteroepitaxial strain relaxation mechanisms and material properties. Particular focus is placed on the interplay between C and Sn incorporation, which enables the formation of crystalline C_y_(Si_z_)Ge_1‐x‐y‐z_Sn_x_ alloys having Sn concentrations reaching 18 at.%.

Building on these advancements, we present controlled epitaxy of high‐quality bulk‐like CGeSn and CSiGeSn alloys, and demonstrate the growth of CGeSn/GeSn multiple quantum wells (MQWs). This expansion of the group‐IV materials palette paves the way to realize innovative device architectures, potentially unlocking novel functionalities for next‐generation semiconductor and photonic applications.

## 2 Results and Discussion

**Figure 1:**
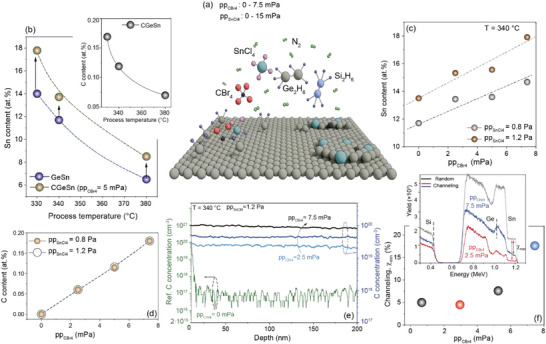
CGeSn Epitaxy: a) Schematic illustration of the epitaxy process and the precursors used. b) Sn content of GeSn alloys as a function of growth temperature, with and without the presence of CBr_4_ in the reactor. Inset: C concentration as a function of growth temperature. c) Sn content in the CGeSn layers as a function of the CBr_4_ partial pressure pp_CBr4_ for two different SnCl_4_ partial pressures at the same growth temperature. d) C concentration versus pp_CBr4_ for the set of layers presented in (c). e) SIMS depth profile of C concentration in CGeSn layers grown at 340 °C at fixed pp_SnCl4_ = 1.2 Pa and different CBr_4_ partial pressures. f) Minimum channeling yield as a function of pp_CBr4_. Inset: the RBS/C spectra for two extreme cases.

The C(Si)GeSn alloys were epitaxially grown on industry‐grade 200 mm Si(100) wafers using a RP‐CVD reactor. The respective precursors employed for the deposition of C, Si, Ge, and Sn were CBr_4_, Si_2_H_6_, Ge_2_H_6_, and SnCl_4_, respectively. A schematic illustration of the epitaxy process is shown in **Figure** **1**a. To address the significant lattice mismatch between the target Ge‐rich alloys and the Si substrate, a 300 nm thick, strain‐relaxed Ge buffer layer was first grown on the substrate. Epitaxial growth of C(Si)GeSn alloy layers was achieved at low deposition temperatures (< 380°C) with Sn incorporation primarily regulated by the growth temperature, as described elsewhere.^[^ ^33^, ^34^, ^35^, ^36^ ^]^ Indeed, for binary GeSn alloy epitaxy, a decreased growth temperature can produce an increase of the Sn incorporation in Ge from 6 at.% to 14 at.%, at respective fixed partial pressures of 12 and 0.8 Pa for the Ge_2_H_6_ and SnCl_4_ precursors (Figure 1b, blue symbols).

To investigate the role of the CBr_4_ precursor, the growth was compared to equivalent growth of binary GeSn in which the partial pressures of Ge_2_H_6_ and SnCl_4_, as well as the reactor total pressure (60 mbar), were kept fixed by adjusting the N_2_ carrier gas going into the reactor.

Interestingly, regardless of the growth temperature, the presence of the CBr_4_ precursor significantly increases Sn incorporation (Figure 1b, gold symbols). At 330°C and a CBr_4_ partial pressure of 5 mPa, the CGeSn alloys achieve Sn concentration of 18 ± 0.5 at.%, as determined by Rutherford backscattering spectrometry (RBS). We note here that such a high Sn concentration could not be achieved in the reactor under any growth conditions, when only the Ge_2_H_6_ and SnCl_4_ precursors^[^ ^37^ ^]^ were employed. This result contrasts with that reported based on MBE growth^[^ ^31^ ^]^ where, using the same CBr_4_ precursor, an 83% decrease in Sn content was observed and underlines the importance of the reaction kinetics in the CVD system. The C content in the layer was measured by calibrated, high‐sensitivity secondary ion mass spectroscopy (SIMS), the results of which are shown in the inset to Figure 1b. Despite the extremely low CBr_4_ partial pressure of just 5 mPa, a noticeable increase in C concentration from 0.07 to 0.17 at.% is observed as the growth temperature is decreased from 380 to 330 °C, mirroring trends seen in CSiGe and CSi epitaxy.^[^ ^38^ ^]^

The impact of the CBr_4_ precursor on the layer growth and the alloy stoichiometry is analyzed at a growth temperature of 340°C for two different SnCl_4_ partial pressures, pp_SnCl4_. For each pp_SnCl4_, the partial pressure of CBr_4_, pp_CBr4_, is varied between 0 and 7.4 mPa (Figure 1c). Increasing the pp_CBr4_ leads to a steady enhancement of Sn incorporation in Ge: from 11.5 to 14.5 at.% at pp_SnCl4_ = 0.8 Pa, and from 13.5 at.% to 18 at.% at pp_SnCl4_ = 1.2 Pa. In the latter case, a maximum Sn concentration of 18 at.% is achieved, but at a higher growth temperature compared to the conditions described above.

The concentration of C in the film is found to be independent of the partial pressure of the SnCl_4_ precursor, and varies linearly with the CBr_4_ partial pressure, ranging from 0.06 to 0.18 at.% for both sets of samples (Figure 1d). The C depth distribution in CGeSn layers grown at a pp_SnCl4_ of 1.2 Pa and varying pp_CBr4_ is presented in Figure 1e, highlighting a homogeneous C distribution throughout the epilayer. Initial insight into the epitaxial quality is obtained from RBS data taken in a channeling orientation. The RBS minimum channeling yield, *χ_min_*, defined as the ratio between the channeling and random signals, reflects the crystalline quality of the layers. The parameter *χ_min_* is shown in Figure 1f, together with the random and channeling RBS spectra for two extremal cases: pp_CBr4_ = 2.5 and 7.5 mPa (inset). For most samples, low *χ_min_* values of ≈5–7% are measured, indicating high crystallinity.^[^ ^39^ ^]^ However, at the highest pp_CBr4_ = 7.5 mPa, *χ_min_* significantly increases to ≈20%, indicating severe degradation of the CGeSn layer's crystallinity. Similar behavior was found by T. Yamaha et al.^[^ ^32^ ^]^ where relatively high C incorporation results in the formation of polycrystalline CGeSn layers.

### 2.1 Lattice‐Strain Evolution in GeSn and CGeSn Layers

The substitution of Ge atoms by larger Sn atoms induces compressive strain, whereas the incorporation of smaller atoms, such as Si or C, introduces tensile strain. By balancing these opposing effects, it is in principle possible to grow lattice‐matched or strain‐compensated C(Si)GeSn epilayers and heterostructures on Ge.

**Figure 2:**
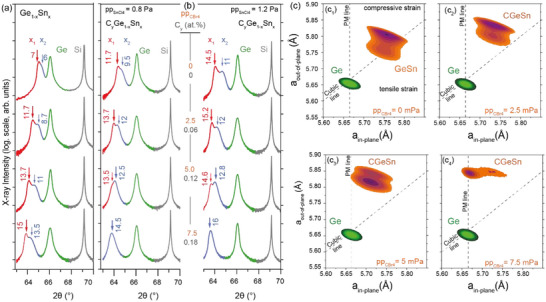
X‐ray analyses: Symmetric 2θ‐ω XRD scans along the (004) plane for a) GeSn_x_ layers with different Sn content, used as comparison reference; b) C_y_Ge_1‐x_Sn_x_ layers grown at a constant temperature of 340 °C for pp_SnCl4_ = 0.8 and 1.2 Pa. The central values indicate the pp_CBr4_ value and the corresponding C concentration in the layers. *x_1_* and *x_2_* denote the Sn content of the two layers formed during growth (marked by arrows) and their relative intensity is marked by the thickness of the arrows. c) Asymmetric XRD‐RSM maps along the (224) crystal plane of CGeSn layers at different values of pp_CBr4_. The diagonal and vertical dotted lines represent the cubic (fully relaxed lattice) and pseudomorphic growth (Ge lattice matched), respectively.

**Figure 3:**
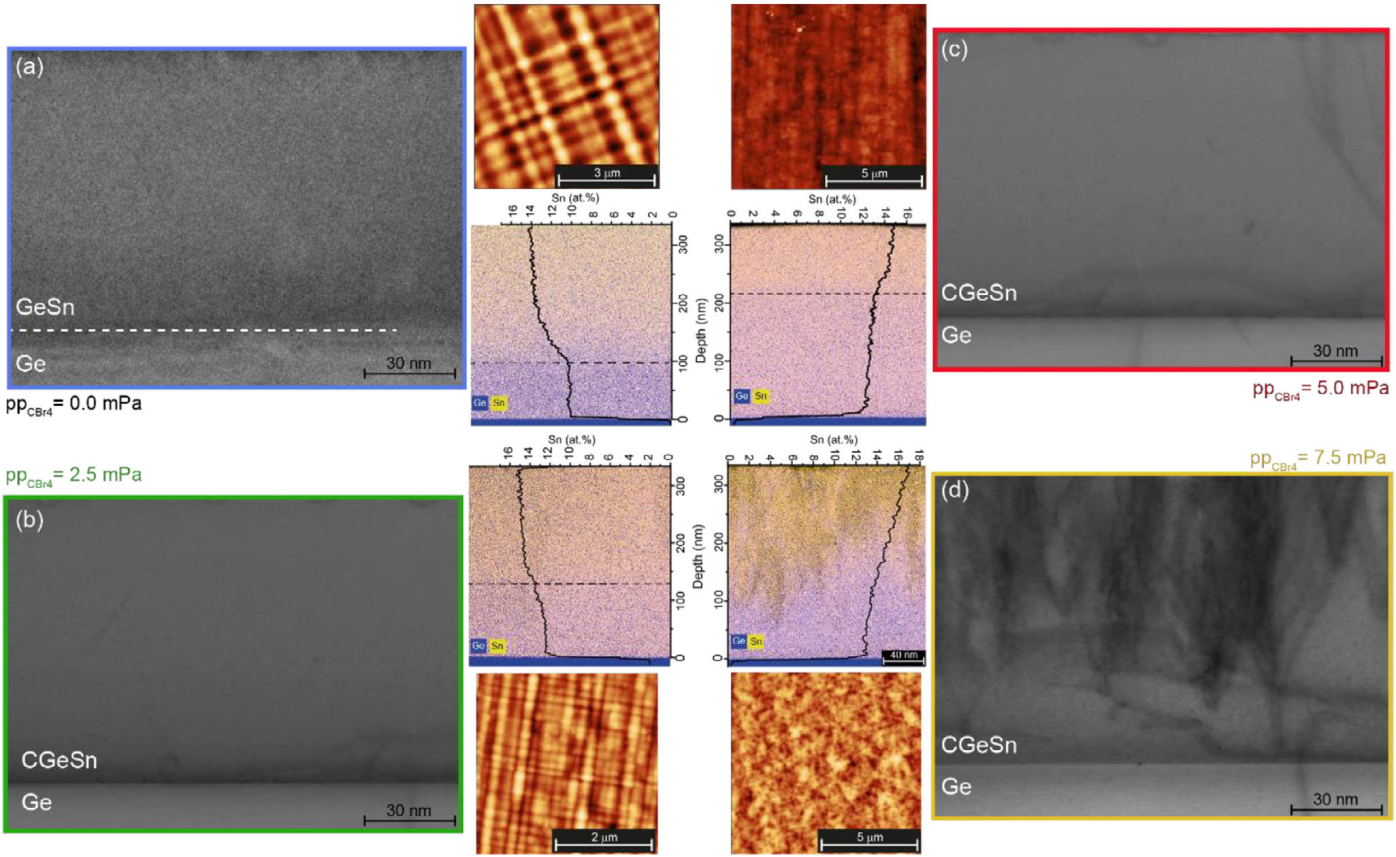
Structural characterization: Structural and morphological characterization of the (C)GeSn samples via HR‐TEM micrographs, including 2D and 1D EDX map and line scans, and surface AFM scans, as a function of pp_CBr4_ during growth.

High‐resolution symmetric 2θ‐ω X‐ray diffraction (XRD) scans along the (004) plane reveal the complex strain landscape in the epitaxial GeSn and CGeSn layers (**Figure** **2**a,b). All epitaxial layers have a total thickness of ∼330‐360 nm, exceeding the critical thickness for strain relaxation, *h_c_*. Two distinct diffraction peaks related to the GeSn layer growth are observed: the first peak corresponds to pseudomorphic growth (Ge_1‐x1_Sn_x1_) that undergoes in‐situ heteroepitaxial strain relaxation after the critical thickness is exceeded, while the second corresponds to the subsequently grown layer possessing higher Sn concentration (Ge_1‐x2_Sn_x2_). The difference in Sn content between the two GeSn layers is ≈1–2 at.% for higher Sn contents. For all binary GeSn alloys, the second (upper) GeSn layer has a larger thickness *h*, indicated by the higher peak intensity of the XRD spectra (thicker arrows in Figure 2a), which scales ≈*h^2^* for a crystalline layer. For the ternary CGeSn alloys, the thickness relation of the two layers changes with the C incorporation. At a C content of 0.12 at.%, the XRD intensity of the two layers reverses, potentially indicating retarded plastic relaxation associated with an increase in *h_c_*. Such behavior was previously observed for CSiGe alloys.^[^ ^40^ ^]^ Further C incorporation results in a single XRD peak. This behavior is consistent with C inhibiting the strain relaxation, discussed below in **Figure** **3**, and is exemplified using 2θ‐ω XRD scans in Figure 2b for the growth conditions presented in Figure 1c.

Additional information is obtained from the asymmetric (224) X‐ray reciprocal space maps (RSM) in Figure 2c. The vertical line corresponds to pseudomorphic growth, i.e., a ‐in general‐ biaxially stressed crystal structure in which the in‐plane lattice parameter matches that of the underlying Ge buffer layer. The cubic line represents the strain‐free (cubic) crystal structure. The Ge buffer layer signal is below the line, indicating a slight biaxial tensile strain, ε_Ge_ = +0.10%, typical of Ge buffer layers grown on Si.^[^ ^41^ ^]^ The (C)GeSn signals are all above the cubic line, indicating biaxial compressive stress. Starting from the position of the Ge_0.855_Sn_0.145_ reference sample, under a −0.3% biaxially compressive strain, the CGeSn in‐plane lattice constant shifts progressively toward the pseudomorphic line with increasing pp_CBr₄_. This corresponds to a continuous build‐up of biaxial compressive strain. For the sample containing 0.12 at.% C, the XRD peak shift is smaller, corresponding to a smaller increase in Sn content after relaxation (see Figure 2b), and their intensity reverses, indicating a larger critical thickness. At a concentration of 0.18 at.% C (pp_CBr4_ = 7.4 mPa), the CGeSn alloy exhibits a single dominant RSM peak, aligned with the fully pseudomorphic condition (≈−2% compressive strain), accompanied by a weak intensity over a broad range of lattice parameters, suggesting significant lattice distortion.

### 2.2 Dislocation Dynamics and Strain Relaxation

When the thickness of a strained epitaxial layer exceeds the critical thickness, it becomes energetically favorable to minimize energy via plastic relaxation, e.g. by forming defects and dislocations.^[^ ^42^, ^43^ ^]^ Similar to the well‐known case of [001] SiGe alloys, here we find that the epilayers relax by generating 60° threading dislocations (TDs). These dislocations glide along the <110> crystal directions on the {111} planes, driven by the lattice stress. As a result, a misfit dislocation (MD) network forms at the (C)GeSn/Ge interface. In SiGe alloys, carbon incorporation is known to introduce localized strain fields around C lattice sites, distorting the lattice structure and raising the Peierls barrier energy, thereby hindering dislocation propagation. In GeSn alloys, strain relaxation predominantly occurs via 90° dislocations, also known as Lomer dislocations.^[^ ^44^ ^]^ When a 60° dislocation encounters a Lomer dislocation on the same slip plane, the interaction typically impedes the 60° dislocation or increases the energy required for it to continue propagating. The increase in carbon concentration results in less efficient strain relaxation, consistent with observations in X‐ray reciprocal space maps showing increased strain retention with higher carbon content (Figure 2c).

This change in dislocation dynamics is clearly evidenced in transmission electron microscopy (TEM) and atomic force microscopy (AFM) measurements (Figure 3). The GeSn reference sample and the lowest C‐content CGeSn layer (0.06 at.% C) display high crystalline quality in the TEM images, with only MDs evident. The cross‐hatch pattern observed via AFM (Figure 3a,b) is a typical surface manifestation of the strain fields caused by these MDs.^[^ ^45^ ^]^ The presence of a uniform cross‐hatch pattern indicates uniform strain relaxation and high‐quality epitaxy.^[^ ^28^ ^]^ Increasing the C content in the epitaxial GeSn layer, the observed TD density increases, and finally, a highly dislocated layer is formed for the highest C‐concentration of 0.18 at.%. As a consequence, the cross‐hatch pattern gradually transitions into a rough‐textured surface, as seen in the AFM images in Figure 3. As discussed below, these defects can act as traps for photoexcited carriers, constituting strong centers for non‐radiative (Shockley‐Read‐Hall) recombination.^[^ ^46^, ^47^ ^]^

The 2D energy‐dispersive X‐ray (EDX) elemental maps, showing the Ge and Sn contents, and the 1D Sn content distribution line scans, confirm the formation of two‐layer elucidated above via XRD: C incorporation acts to limit strain relaxation, increasing the critical thickness, an effect that is observable even at an ultra‐dilute C content of 0.06 at.% (Figure 3b), and also in thicker layers having C content 0.12 at.% C (Figure 3c).

These CGeSn epitaxial growth experiments yield four key conclusions: i) an increase in CBr_4_ partial pressure leads to higher concentrations of both Sn and C, with the effect being more pronounced for Sn; ii) C incorporation in ternary CGeSn alloys depends on the growth temperature and the CBr_4_ partial pressure, but is largely independent of SnCl_4_ partial pressure; iii) the crystalline quality of the epitaxial layers rapidly degrades with increasing C content due to dislocation pinning. This approach provides a novel method to tune Sn content at constant growth temperature, where the other precursor parameters have already been optimized.

### 2.3 Theoretical Band Structure Consideration and Optical Properties

Detailed understanding of the electronic structure of highly‐mismatched group‐IV dilute carbide alloys, including binary CGe, is not yet extensive, and is completely lacking for ternary CGeSn and quaternary CSiGeSn alloys. For group‐IV‐based lasers, incorporation of C has been proposed as a solution to enable room‐temperature operation of electrically pumped GeSn lasers.^[^ ^48^ ^]^ However, this proposal relies on one of two competing descriptions of the electronic and optical properties of Ge_1‐x_C_x_ alloys. These analyses of the Ge_1‐x_C_x_ electronic structure can be understood by analogy to the highly‐mismatched III‐V dilute nitride alloys GaN_x_As_1‐x_ and GaN_x_P_1‐x_.^[^ ^49^, ^50^ ^]^ Based on calculations for idealized ordered alloys, Stephenson et al.^[^ ^51^, ^52^ ^]^ proposed that substitutional C in Ge acts like N in GaAs, producing a localized state above the Γ‐point of the conduction band (CB) edge. This localized C state undergoes a band‐anticrossing (BAC) interaction with the Γ‐valley CB edge of the Ge host matrix, producing a direct band gap by pushing a primarily Ge‐derived state downward in energy.^[^ ^23^ ^]^ This, in turn, is expected to lead to a high radiative recombination rate since the alloy CB minimum is then derived primarily from the Ge Γ‐valley CB edge, which has a high optical matrix element with the comparatively unperturbed Ge_1‐x_C_x_ alloy valence band (VB) maximum. Conversely, Broderick et al.^[^ ^53^ ^]^ predicted that the Ge_1‐x_C_x_ electronic structure is akin to that of GaN_x_P_1‐x_. In this case, the CB minimum in an ordered Ge_1‐x_C_x_ alloy is primarily comprised of a linear combination of indirect Ge L‐point minimum states, possessing a low optical matrix element with the VB maximum, and hence producing a “pseudo‐direct” bandgap with a low radiative recombination rate. This interpretation is consistent with the pressure‐dependent calculations of Kirwan et al.^[^ ^54^ ^]^ Furthermore, by considering realistic disordered alloys, Broderick et al.^[^ ^53^ ^]^ showed that the optical transition strength associated with the direct bandgap is significantly degraded in the presence of short‐range C‐related alloy disorder. In this case, C incorporation produces a distribution of localized impurity states within the Ge bandgap, with the degree of impurity state localization increasing for larger clusters of neighboring C atoms, whose associated localized states lie energetically deeper within the Ge bandgap. These C‐related localized states hybridize strongly with the Γ‐valley CB edge state, distributing the Γ‐valley CB edge character across a large number of localized states in a disordered Ge_1‐x_C_x_ alloy. This results in no single alloy CB state having a large optical matrix element for transitions to the VB edge, thereby suppressing the intensity of optical emission from C‐containing alloy samples. This strong C‐induced hybridization distributes Γ‐valley CB edge character across a multiplicity of alloy states of differing energy, driving strong inhomogeneous spectral broadening of the optical emission. The measured PL from C‐containing alloys is thus predicted to be markedly suppressed and strongly inhomogeneously broadened. This, combined with the potential that deep C‐related defects could act as centers for non‐radiative Shockley‐Read‐Hall recombination, suggests that C incorporation can degrade optical emission in the presence of significant C clustering.^[^ ^53^ ^]^

**Figure 4:**
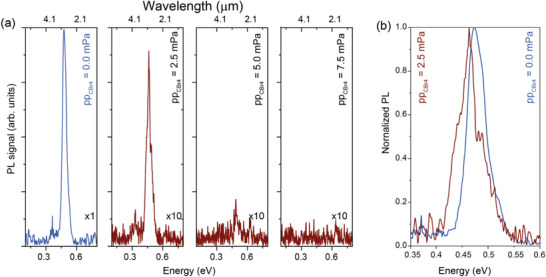
Optical study: a) PL emission from (C)GeSn layers grown at different CBr_4_ partial pressures. For clarity, the same y‐scale is used, and an amplification factor for the peak PL intensities has been used. b) Normalized PL emission of the GeSn (blue) and CGeSn (red) samples of (a).

Experimentally, **Figure** **4**a compares the photoluminescence (PL) emission spectra at 77 K of GeSn and CGeSn epilayers. The introduction of dilute amounts of C in bulk GeSn alloys strongly suppresses optical emission, showing a strong decrease in the PL intensity with increasing C content. A redshift of 11 meV is observed between the binary GeSn alloy and the ternary CGeSn alloy containing only 0.06 at.% C (Figure 4b). This energy shift is in line with the expected bandgap reduction of GeSn, due to an increase in Sn content driven by the presence of the C precursor CBr_4_. This is in line with aspects of the theoretical predictions of both Stephenson et al.^[^ ^51^, ^52^ ^]^ and Broderick et al.^[^ ^53^ ^]^ The sharp reduction in PL intensity, even at ultra‐dilute C content, is consistent with the analysis and predictions of Broderick et al.^[^ ^53^ ^]^ This suggests that a degradation of the optical emission may be an intrinsic consequence of the impact of C incorporation on the alloy electronic structure, and is not directly attributable due to poor crystalline quality.

The primary challenge in CGeSn epitaxy is achieving semiconductors with high crystalline quality. This suggests that it may be beneficial to pursue the growth of strain‐compensated heterostructures, thereby eliminating the need for strain relaxation. Two approaches can be considered in this regard:

i) Intrinsic strain engineering: This involves adding an element that adjusts the lattice parameter, balancing the effects of C and Sn strain, e.g. via co‐incorporation of Si.
ii) Extrinsic strain engineering: Instead of growing CGeSn alloys directly on Ge, they can be grown on GeSn relaxed buffers, reducing the stress experienced by the lattice. This approach also allows for the deposition of an intermediate high Sn‐content layer to enable strain balancing in MQW structures, facilitating the growth of arbitrarily thick active layers.

### 2.4 Intrinsic Strain Engineering: Quaternary CSiGeSn Alloys

Epitaxial growth of semiconductor heterostructures based on group‐IV elements is driven by the potential to introduce new functionalities to the existing CMOS platform, along with the significant opportunity to produce cost‐effective group‐IV integrated circuits in high‐volume commercial Si foundries. The group‐IV semiconductor alloy palette spans from binary SiGe or GeSn to ternary CGeSn and SiGeSn, ultimately reaching the quaternary CSiGeSn system. While real‐world challenges including solubility, segregation, and stability must be addressed in practical epitaxy, theoretical predictions suggest that these alloys could exhibit a direct bandgap ranging from 1.5 to −0.4 eV.^[^ ^26^ ^]^

**Figure 5:**
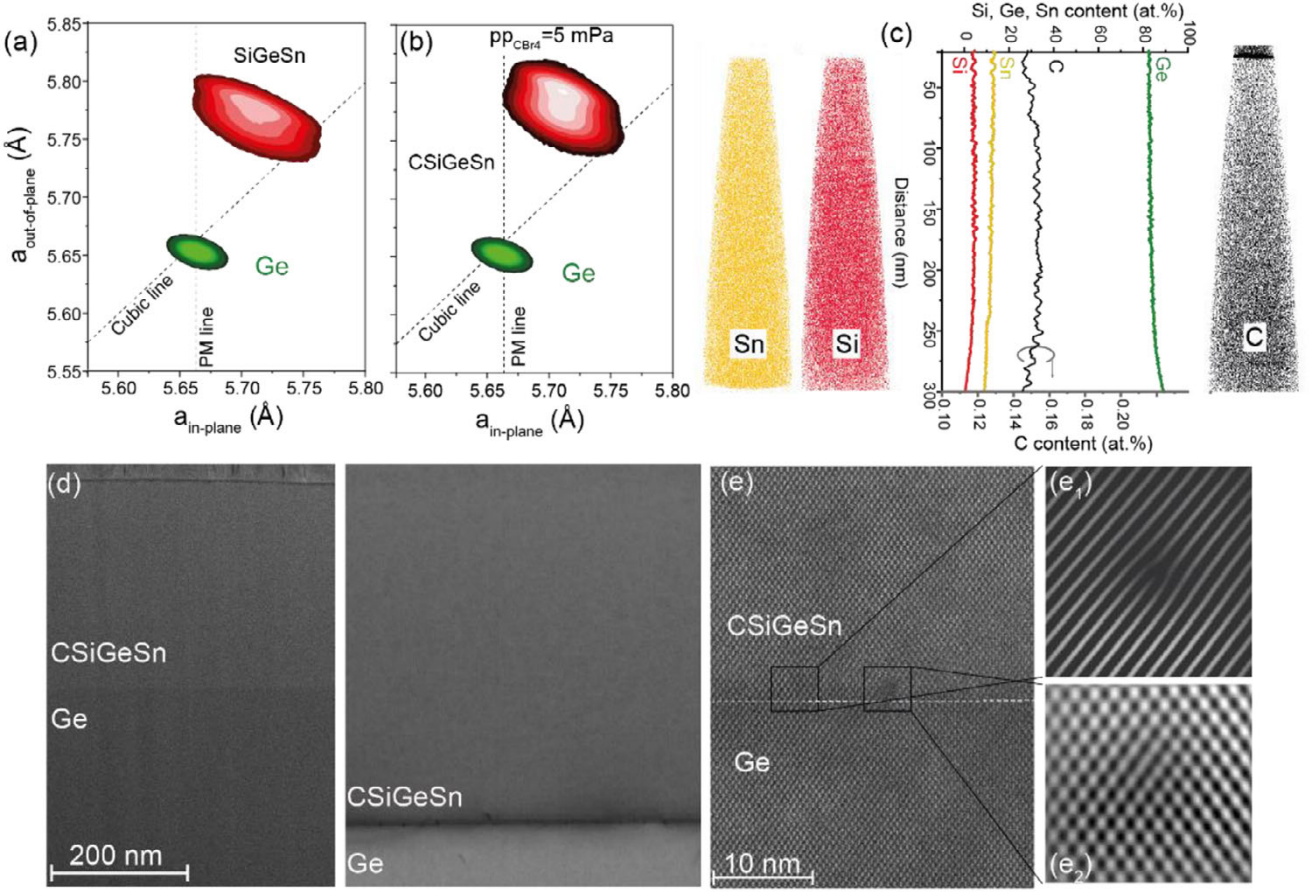
Quaternary CSiGeSn alloys: Asymmetric XRD‐RSM along the (224) crystal plane of a) ternary SiGeSn and b) quaternary CSiGeSn layers. c) 3D APT data of CSiGeSn layer, and the line profile of Si, Ge, and Sn content, and the SIMS depth profile of C content. A uniform distribution of all group‐IV elements is present in the layer. d,e) HR‐TEM micrographs of a CSiGeSn layer at different magnifications. Insets e_1_,e_2_: Lomer dislocations where a {111} plane is missing.

Following the growth methodology described above and elsewhere,^[^ ^29^ ^]^ ternary SiGeSn and quaternary CSiGeSn thin epitaxial layers have been grown. As for the case of binary GeSn, the introduction of CBr_4_ drives C incorporation and enables epitaxial growth of quaternary CSiGeSn alloys. A comparison of the XRD‐RSM spectra of 300 nm thick SiGeSn and CSiGeSn layers, grown under similar conditions, is presented in **Figure** **5**a,b. By introducing CBr_4_ with pp_CBr4_ = 5 mPa, the epitaxial Si_0.05_Ge_0.83_Sn_0.12_ layer changes its stoichiometry by increasing its Sn content and C incorporation. The XRD‐RSM peak shifts upward, indicating a slight increase in strain in the layer, while the lattice strain relaxation remains almost constant at ≈75%. Distinct from the epitaxy of ternary CGeSn under the same growth conditions, the enhancement in Sn content is slightly reduced by ≈1 at.%. This may be attributed to the role of Si atoms in lowering surface energy and influencing diffusion kinetics during epitaxy. Atom probe tomography (APT) data show a uniform 3D distribution of the constituent group‐IV atoms, without any indication of C clustering or Sn segregation (Figure 5c). The line‐depth profile enables the determination of the atomic concentrations of Si, Ge, and Sn (upper scale), while the C concentration (lower scale) of 0.15 at.% C is determined via SIMS using C calibration samples. The high crystallinity of the layer is confirmed by HR‐TEM images, shown in Figure 5d,e, which also reveal the presence of a Lomer dislocation, where a {111} plane is missing. No cross‐layer threading dislocations are observed in the investigated sample area, and a sharp interface is visible between the quaternary CSiGeSn layer and the Ge buffer layer, the latter of which contains misfit dislocations. Si incorporation compensates for the increased defect densities resulting from the enhancement of Sn, due to the introduction of CBr_4_ in the growth process.

This growth and characterization study presents comprehensive experimental evidence of the epitaxial growth of a quaternary thin film containing the group‐IV elements C, Si, Ge, and Sn. This achievement pioneers a novel approach to engineer and enhance the electronic and optical properties of group‐IV alloys, paving the way for advanced device development.

### 2.5 Extrinsic Strain Engineering: Advanced CGeSn/GeSn MQWs Heterostructures

**Figure 6:**
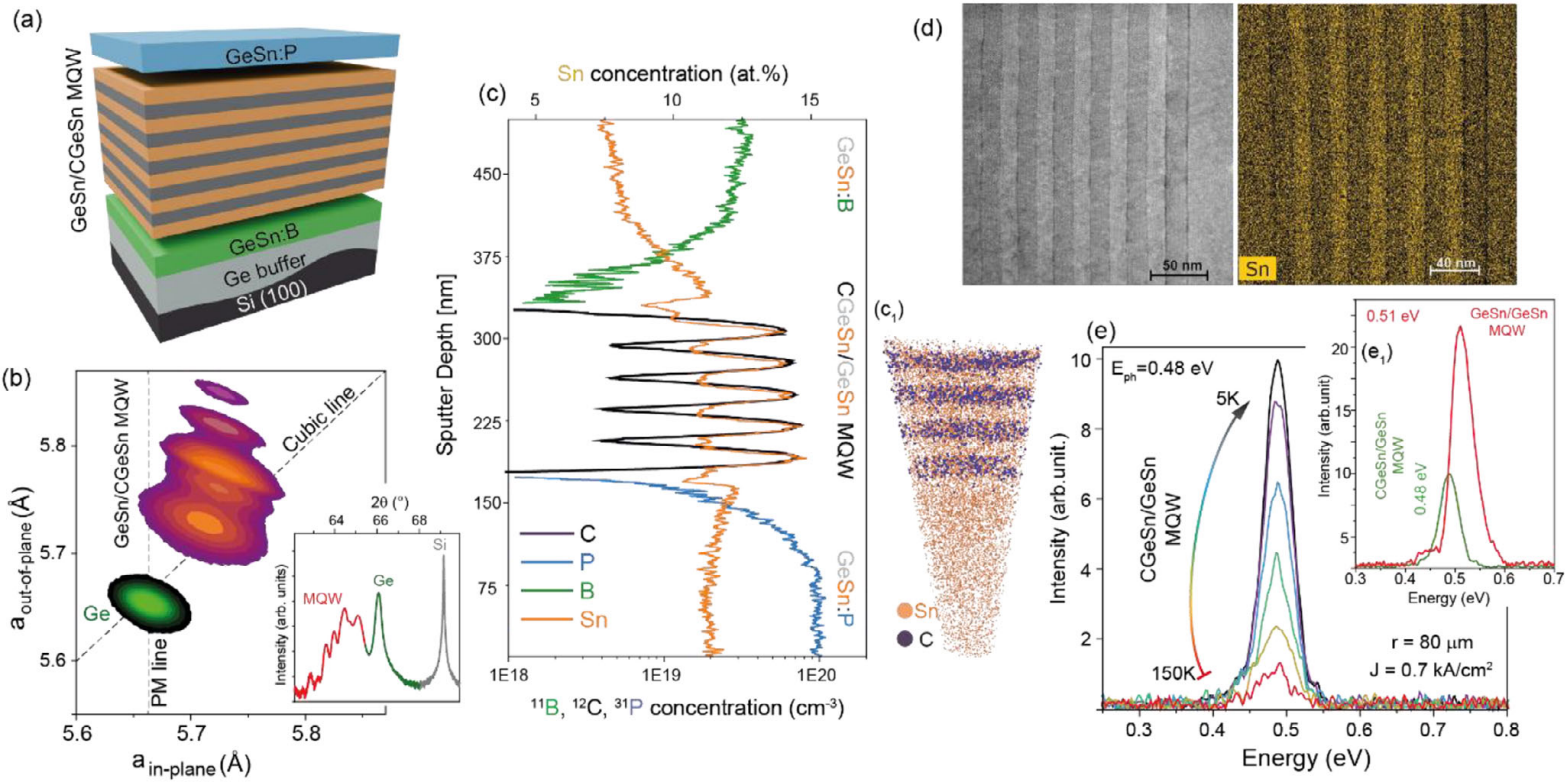
CGeSn/GeSn MQWs heterostructures: a) Schematic 3D image of the CGeSn/GeSn MQW heterostructure. b) Asymmetric RSM and (inset) symmetric 2θ‐ω XRD scans showing oscillation fringes, which indicate high crystalline quality and well‐defined well/barrier interfaces. c) SIMS elemental depth spectra and (c_1_) 3D map of C and Sn elements by APT, indicating C incorporation only in the well layers. d) Cross‐sectional TEM image and corresponding EDX map showing the Sn concentration difference between the wells and barrier layers. e) Temperature‐dependent EL spectra for the 80 µm radius micro‐disk LED. e_1_) EL comparison at 5K of GeSn/GeSn and CGeSn/GeSn MQWs grown under similar conditions.

An alternative approach to avoid large strain build‐up in a ternary CGeSn layer is to employ growth on a strain‐relaxed GeSn buffer. This approach is typically used in the growth of the strain‐relaxed GeSn/SiGeSn MQWs, as employed in optically^[^ ^55^ ^]^ and electrically pumped lasers.^[^ ^17^ ^]^ Several sets of 5×{14 nm CGeSn well/17 nm GeSn barrier} MQWs have been grown on top of a 300 nm boron‐doped Ge_0.93_Sn_0.07_ buffer layer, which is used as the bottom contact of a vertical light‐emitting MQW diode (**Figure** **6**a). The well/barrier composition profile is obtained by decreasing/increasing the N_2_ carrier gas flow, while keeping the deposition temperature constant.^[^ ^37^ ^]^ The CBr_4_ precursor is introduced during the growth of the wells and is expected to further increase the Sn content, thereby reducing the well bandgap to produce type‐I CGeSn/GeSn band alignment, confining both electrons and holes in the CGeSn wells. The stack is completed with a highly phosphorus‐doped Ge_0.89_Sn_0.11_ layer as a top contact.

As can be observed in the (224) XRD‐RSM of Figure 6b, the GeSn buffer layer is highly relaxed (≈85%), and the CGeSn/GeSn MQWs are pseudomorphically grown on top of it. The appearance of satellite peaks indicates the presence of smooth CGeSn/GeSn interfaces, and high crystallinity in the MQW structures. The elemental composition and atomic distribution of each structure were respectively investigated by SIMS and APT (Figure 6c). C is found to be present only in the CGeSn well layers, and no indications of C clustering or Sn segregation were evident in the APT data. The high crystalline quality is evidenced by cross‐sectional TEM micrography (Figure 6d), while corresponding EDX mapping reveals the Sn concentration difference between the well and barrier layers.

The optical quality of the MQW structures was investigated via electroluminescence (EL) measurements. Vertical micro‐disk LEDs were fabricated using standard Si technology (see Ref. ^[^ ^17^ ^]^ for details). The temperature‐dependent EL spectra up to 150 K from a diode of 80 µm radius, for a current density of 0.7 kA cm^−2^, are shown in Figure 6e. The peak EL emission lies in the near‐infrared, centred at a wavelength of 2.54 µm at 5 K. For comparison, a LED based on a similar C‐free GeSn/GeSn heterostructure, was fabricated. For that reference structure, the Sn content was determined to be 2 at.% lower than in the CGeSn/GeSn structure, when CBr_4_ was introduced during the growth of the C‐containing quantum wells. This difference in Sn content results in a blueshift of the LED emission wavelength in the reference structure (Figure 6e inset). However, the EL intensity of the CGeSn MQW is reduced by ≈50% vs. that from the reference C‐free GeSn MQW. This is consistent with the reduction in emission intensity observed in our PL measurements on bulk‐like ternary CGeSn epilayers (Figure 4a), but note that the extent of this reduction is well mitigated in the MQW structures. The optical emission spectra from CGeSn bulk (Figure 4) and MQW heterostructures (Figure 6) provide evidence of the fundamental zone‐center CB‐VB transition in direct bandgap CGeSn alloys. Following this proof‐of‐principle demonstration of optical emission, more detailed experimental data – e.g. on the impact of varying C concentrations and QW thickness – are required to confirm the potential of this new class of direct bandgap group‐IV heterostructure, and to investigate the hypothesis that C can act as a source of non‐radiative recombination.

These findings significantly expand the design space for direct bandgap group‐IV heterostructures. To our knowledge, this constitutes the first demonstration of growth of a quaternary group‐IV alloy containing all of C, Si, Ge, and Sn. Ongoing material characterization will be crucial for understanding the properties of Group IV alloys and for developing innovative methods to tailor and enhance their electronic and optical performance, ultimately accelerating progress in advanced semiconductor technologies.

## 3 Conclusion

Using an industry‐standard CVD reactor, high‐quality epitaxial growth of CMOS‐compatible all‐group‐IV semiconductors was demonstrated. Incorporating CBr_4_ as a C precursor in the established direct bandgap GeSn system allows to realize ternary CGeSn alloys. The addition of CBr_4_ to Ge_2_H_6_ and SnCl_4_ precursors in an inert N_2_ atmosphere was found to enhance Sn incorporation in Ge, offering a novel route to achieve high‐Sn content at increased growth temperature, opening a new pathway to improve crystalline quality in all group‐IV epitaxy. In strained layers, C incorporation suppresses dislocation migration and delays plastic strain relaxation by increasing critical thickness, leading to reduced crystalline quality that limits the potential of bulk CGeSn for optoelectronic applications. However, this challenge can be addressed by growing CGeSn/GeSn MQW heterostructures on a strain‐relaxed GeSn buffer layer. These heterostructures demonstrate high crystal quality, and their potential is confirmed via the demonstration of near‐infrared emission at 2.54 µm in prototype LEDs fabricated from these advanced all‐group‐IV heterostructures. Furthermore, we demonstrate that co‐alloying Si, via a Si_2_H_6_ precursor, mitigates the formation of C‐induced crystalline defects, enabling the growth of high‐quality quaternary CSiGeSn semiconductor alloys.

To our knowledge, this study is the first to report epitaxial growth of an all‐group‐IV CSiGeSn alloy, as well as direct bandgap CGeSn‐based MQW heterostructures. Having established the growth of these new direct bandgap group‐IV semiconductors, further research is required to understand their properties and potential applications. Despite identified challenges, our results highlight a promising path toward monolithic integration of all‐group‐IV semiconductors.

## 4 Experimental Section

The Sn content, thickness, and crystalline quality of the C(Si)GeSn films were extracted by fitting the random/channeling Rutherford backscattering spectroscopy (RBS) spectra using a Tandetron accelerator with 1.4 MeV He+ ion beam. Calibrated secondary ion mass spectrometry (SIMS, ION‐TOF 5 system) and atom probe tomography (APT) were employed to determine Si, Sn, and C elemental concentration and spatial distribution through the thin epitaxial film. The stoichiometry values were used to extract the strain values through X‐ray diffraction reciprocal space maps (XRD‐RSM) measurements using a Rigaku SmartLab diffractometer. Atomic Force Microscopy (AFM, Bruker Veeco Dimension Icon) was used to reveal the surface morphology and roughness of the grown layers. The crystal structure and the atomic arrangement were accessed via FEI Titan 80–300 high‐resolution transmission electron microscopy (HR‐TEM). Scanning TEM‐EDX measurements were carried out using a 200 keV FEI Tecnai Osiris system. The photoluminescence (PL) signal from the samples was collected perpendicular to their surfaces using a commercial mid‐infrared optical microscope from Bruker. The signal was then analyzed with an Invenio‐R Fourier transform infrared (FTIR) spectrometer. Detection was carried out using an N_2_‐cooled InSb photodetector. Prior to measurements, thorough spectral calibration of the spectrometer, photodetectors, and internal source was performed. Electroluminescence (EL) measurements were done in a Bruker VERTEX 80 v FT‐IR spectrometer, and their light emission was analyzed using a Fourier‐transform spectrometer in step‐scan mode, which detects the modulated signal intensity with a lock‐in amplifier to achieve a higher signal‐to‐noise ratio and, hence, better visibility for low‐intensity signals.

## Conflict of Interest

The authors declare no conflict of interest.

## Data Availability

The data that support the findings of this study are available from the corresponding author upon reasonable request.
